# Clinical experience of volumetric‐modulated flattening filter free stereotactic body radiation therapy of lesions in the lung with deep inspiration breath‐hold

**DOI:** 10.1002/acm2.13733

**Published:** 2022-07-22

**Authors:** Siri T. Mørkeset, Christoffer Lervåg, Jo‐Åsmund Lund, Christer Jensen

**Affiliations:** ^1^ Department of Oncology and Rehabilitation Møre and Romsdal Hospital Trust Ålesund Hospital Ålesund Norway; ^2^ Department of Health Sciences in Ålesund Faculty of Medicine and Health Sciences Norwegian University of Science and Technology (NTNU) Ålesund Norway; ^3^ Department of Medicine and Healthcare Møre and Romsdal Hospital Trust Ålesund Hospital Ålesund Norway

**Keywords:** deep inspiration breath‐hold, DIBH, free breathing, lung, radiation therapy, radiotherapy, RT, SBRT, stereotactic body radiotherapy

## Abstract

This clinical study aimed to evaluate lung cancer patients’ ability to perform deep inspiration breath‐hold (DIBH) during CT simulation and throughout the treatment course of stereotactic body radiation therapy (SBRT). In addition, target sizes, organ at risk (OAR) sizes, and doses to the respective volumes in filter‐free volumetric‐modulated arc therapy plans performed under free‐breathing (FB) and DIBH conditions were evaluated. Twenty‐one patients with peripheral lesions were included, of which 13 were eligible for SBRT. All patients underwent training for breath‐hold during CT, and if they complied with the requirements, two CT scans were obtained: CT scan in DIBH and a four‐dimensional CT scan in FB. The treatment plans in FB and DIBH were generated, and the dose parameters and volume sizes were compared. The endpoints for evaluation were patient compliance, target dose coverage, and doses to the OARs. This clinical study showed high patient DIBH compliance during both CT simulation and treatment for patients with lung cancer. A significant reduction in target volumes was achieved with SBRT in DIBH, in addition to significantly decreased doses to the heart, chest wall, and lungs. DIBH in SBRT of lung lesions is feasible, and a routine to manage intra‐fractional deviation should be established upon implementation.

## 1 INTRODUCTION

Stereotactic body radiation therapy (SBRT) plays an important role in the treatment of inoperable early‐stage non‐small cell lung cancer (NSCLC).^1^ Lung cancer is the leading cause of cancer‐related death among both men and women globally, and NSCLC is associated with poor 5‐year relative survival rates of approximately 30% and 65% for regional and localized disease, respectively.^2^ NSCLC accounts for approximately 85% of all lung cancers.^3^ Since lung cancer can be categorized into several subgroups based on the morphology and extent of disease, the prognosis and treatment of choice differ across subgroups. Improvements in lung cancer treatment over the past two decades may have contributed to the recent increase in the survival rate.^2^

With the rapid advancements in RT, patients with stage I‐III disease are being offered SBRT with a curative intent.^3^ Hansen et al. reported that the overall survival in 544 inoperable patients with early NSCLC treated with SBRT was 43.9 months, and the 5‐year OS was the highest (45%) for patients under 70 years of age.^4^ Pooled data from two independent randomized phase 3 trials of SBRT in patients with operable stage I NSCLC versus lobectomy demonstrated 3‐year survival rates of 95% versus 79% in the SBRT and lobectomy groups, respectively.^5^ These studies show that a number of patients survive for longer periods of time and may experience detrimental long‐ as well as short‐term effects of RT.

The standard RT technique for the treatment of lung lesions is performed while the patient is breathing freely; thus, the target is moving during treatment. Seppenwoolde et al.^6^ found that lesions in the lower lobe, close to the diaphragm, move 12 ± 2 mm in the craniocaudal direction as a result of respiratory motion. Treatment of moving targets is challenging, and the movements can also cause geometrical distortions in the computed tomography (CT) images obtained for treatment planning.^7^ Traditionally, large margins have been applied to account for lesion motion, but the adoption of four‐dimensional CT (4DCT) has recently improved target delineation owing to its ability to provide information on lesion movement.^8^ 4DCT in free‐breathing (FB) mode is now recommended in guidelines and ensures coverage of the whole tumor in each position during the breathing cycle.^9^

Deep inspiration breath‐hold (DIBH) treatment has been introduced for left‐sided breast cancer in the last decade, and this technique has been described to be beneficial in breast cancer RT.^10^ Gated treatment with DIBH has been shown to offer several advantages for RT in lung lesions with conventional fractionation, since it can reduce doses to healthy tissue,^11^, ^12^, ^13^ improve image quality,^14^ and increase dose conformity.^13^ Despite these findings, however, DIBH is currently not the preferred technique for treating lung cancer.

Treatment equipment and techniques have evolved recently, resulting in shorter beam‐on‐time and more conforming dose distributions. The use of modern linear accelerators equipped with flattening filter‐free (FFF)^15^ delivery and treatment techniques such as volumetric‐modulated arc therapy (VMAT) has considerably shortened treatment times for SBRT.^16^ Moreover, SBRT offers the advantage of allowing only a few fractions over the treatment course with superior local control and toxicity rates.^17^ These new techniques, adapted in modern RT, may contribute to a tolerable breath‐hold treatment for this group of patients. SBRT might become the treatment technique of choice for treating smaller lung lesions in the future.

This study aimed to evaluate whether lung cancer patients receiving treatment for a lesion in the lung could endure DIBH during CT training and throughout the SBRT treatment course. In addition to evaluating the ability of these patients to hold their breath, this study aimed to evaluate the sizes and doses to the targets and organs at risk (OAR) by comparing treatment plans in FB and DIBH with the FFF VMAT technique.

## 2 METHODS

### 2.1 Patient selection and training

Patients with lung cancer referred to Ålesund Hospital between April 2020 and February 2021 were consecutively recruited. Written informed consent was obtained from 21 lung cancer patients referred for radiotherapy of locally advanced NSCLC; 13 of them had GTV diameter <6 cm and were treated with SBRT and included in this evaluation. This study was approved by the regional ethics committee. All patients had Eastern Co‐operative Oncology Group (ECOG) performance status ≤2.^18^ Median age was 74 years (range 56–86). All patients were immobilized using WingSTEP (IT‐V, Innsbruck, Austria) and ProSTEP (Elekta, Stockholm, Sweden). CT was performed with a Brilliance Big Bore Oncology (Philips, Amsterdam, Netherlands), and breathing was registered using Sentinel (C‐RAD, Uppsala, Sweden). All patients underwent breath‐hold training before CT, including deep breathing to determine their maximum amplitude level.

To be eligible for the DIBH treatment, patients had to hold their breath multiple times; each breath‐hold lasted a minimum of 20 s for 180 s in total. Patients who met these requirements underwent CT in DIBH in addition to FB in 4DCT. The amplitude level was established at a minimum of 80% of the maximum inhalation, and the window of the amplitude was set to 3 mm. The slice thickness was 2 mm. Overall, 20 of 21 patients complied with the requirements, of which 13 were also eligible for SBRT treatment; three had two separate lesions, resulting in 16 different targets.

### 2.2 Target and OAR delineation

Target and OAR delineation was performed using RayStation version 9A (RaySearch Laboratories, Stockholm, Sweden). The oncologists delineated the gross tumor volume (GTV),^19^ heart, and esophagus. The clinical target volume (CTV)^19^ was derived as a uniform extension of 5 mm from the GTV in all directions. The planning target volume (PTV) was then derived as an extension of 5 mm from the CTV in all directions.^19^ Radiation therapists used a delineation script for OARs and performed quality assurance for all generated volumes. The same individual delineated the volumes in both image sets for each patient, avoiding inter‐observer variability.

### 2.3 Treatment planning and delivery

Conformal SBRT VMAT plans were generated in RayStation by using two arcs on a modeled Elekta VersaHD with 5‐mm multileaf collimators. The prescription dose was D_99%_ to the PTV, and it was administered using only a 6‐MV FFF beam due to the ability of this beam to deliver high doses with a short beam‐on time. The dose was calculated with a collapsed cone v5.1 algorithm and a dose grid of 0.2 × 0.2 × 0.2 cm^3^.

An in‐house protocol with the clinical goals listed in Supplement 1 was used during the treatment planning. SBRT dose limits were based on the findings of previous studies.^3^, ^20^ The conformity index (CI) was calculated in RayStation and defined as the ratio between the PTV volume covered by the 100% isodose and the total 100% isodose volume.

All patients were treated with catalyst (C‐RAD, Uppsala, Sweden) breathing control on Elekta VersaHD (Elekta, Stockholm, Sweden) machines. The patients underwent two cone‐beam CT (CBCT) examinations before each fraction with action limits to verify positioning. The tumor match was always the decisive factor and couch movements followed all CBCTs; the first and second CBCT examinations had an action limit of 5/3 mm in all directions, as well as a limit of 3° rotational deviation.

### 2.4 Statistics

To compare the two techniques, data distribution was assessed.^21^ Data were not normally distributed and were analyzed using the Wilcoxon signed‐rank test for statistical analysis in SPSS version 27 (IBM, Armonk, US). Results were considered significant when *p* < 0.05.

## 3 RESULTS

In this study, 20 of 21 patients were able to comply with the DIBH requirements, but only 13 were candidates for SBRT. One patient was unable to hold his breath at all, and his training was terminated. All DIBH SBRT treatments were performed in a 20‐min time slot. The mean amplitude was 11 mm, and the mean maximum breath‐hold was 41 s. The mean estimated beam‐on time, calculated in RayStation, for FB and DIBH was 168 and 155 s, respectively.

### 3.1 Target size

**FIGURE 1:**
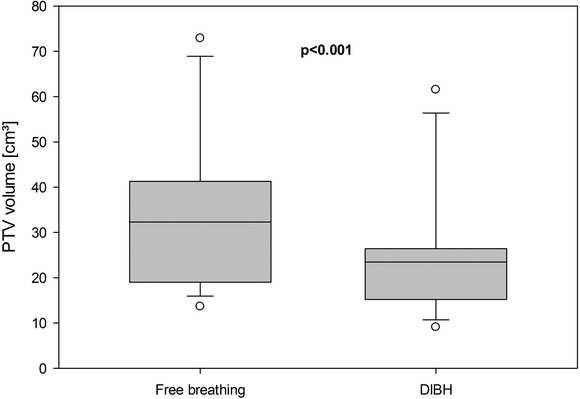
Planning target volume (PTV) volume in free breathing and deep inspiration breath‐hold (DIBH). Boxes extending from the 25th to the 75th percentiles. The whiskers represent 10th and 90th percentiles, and all outliers are displayed

A significant difference between FB and DIBH with regard to overall PTV volumes and DIBH with smaller volumes was observed (Table 1 and Figure 1).

**TABLE 1:** Dosimetric comparison of free breathing and DIBH in VMAT‐plans

	FB		DIBH			
Parameter	Median	Range	Median	Range	Number of volumes	*p*‐Value
Target						
PTV D_98%_ (Gy)	45.61	31.72–55.86	54.28	36.58–55.86	16	0.86
OAR						
Clinical maximum dose (Gy)	68.38	52.47–76.24	67.11	56.46–76.69	13	0.97
Lungs‐GTV mean (Gy)	3.71	0.66–6.35	2.64	0.49–5.50	13	<0.01
Heart mean (Gy)	0.65	0.07–3.37	0.34	0.03–1.73	13	<0.01
Heart D_2%_ (Gy)	5.77	0.32–16.98	3.46	0.12–8.27	13	<0.01
Spinal canal D_2%_ (Gy)	7.72	3.58–12.69	8.23	3.89–12.04	13	0.20
Esophagus D_5cc_ (Gy)	6.31	0.81–12.28	4.68	0.12–10.72	13	0.08
Esophagus D_0,00cc_ (Gy)	12.37	3.50–17.52	10.04	0.22–16.69	13	0.10

Abbreviations: CTV, clinical target volume; D_0,00cc_, maximum dose administered to a 0.00‐cm^3^ volume; D_2%_, maximum dose administered to 2% of volume; D_2cc_, maximum dose administered to a 2‐cm^3^ volume; D_5cc_, maximum dose administered to a 5‐cm^3^ volume; D_98%_, dose to 98% of the target volume; DIBH, deep inspiration breath‐hold; FB, free breathing; GTV, gross tumor volume; OAR, organs at risk; PTV, planning target volume; VMAT, volumetric‐modulated arc therapy.

### 3.2 Dosimetric parameters

**FIGURE 2:**
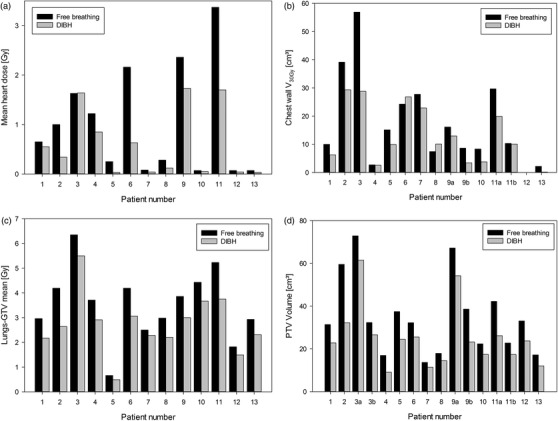
Comparison of free breathing (black bars) and deep inspiration breath‐hold (gray bars) in heart (a), chest wall (b), lungs‐gross tumor volume (GTV) (c) and planning target volume (PTV) (d). Patients 9 and 11 had doses to chest wall in both left and right lung due to them having targets in both lungs, while patient 3 had two targets in one lung

**FIGURE 3:**
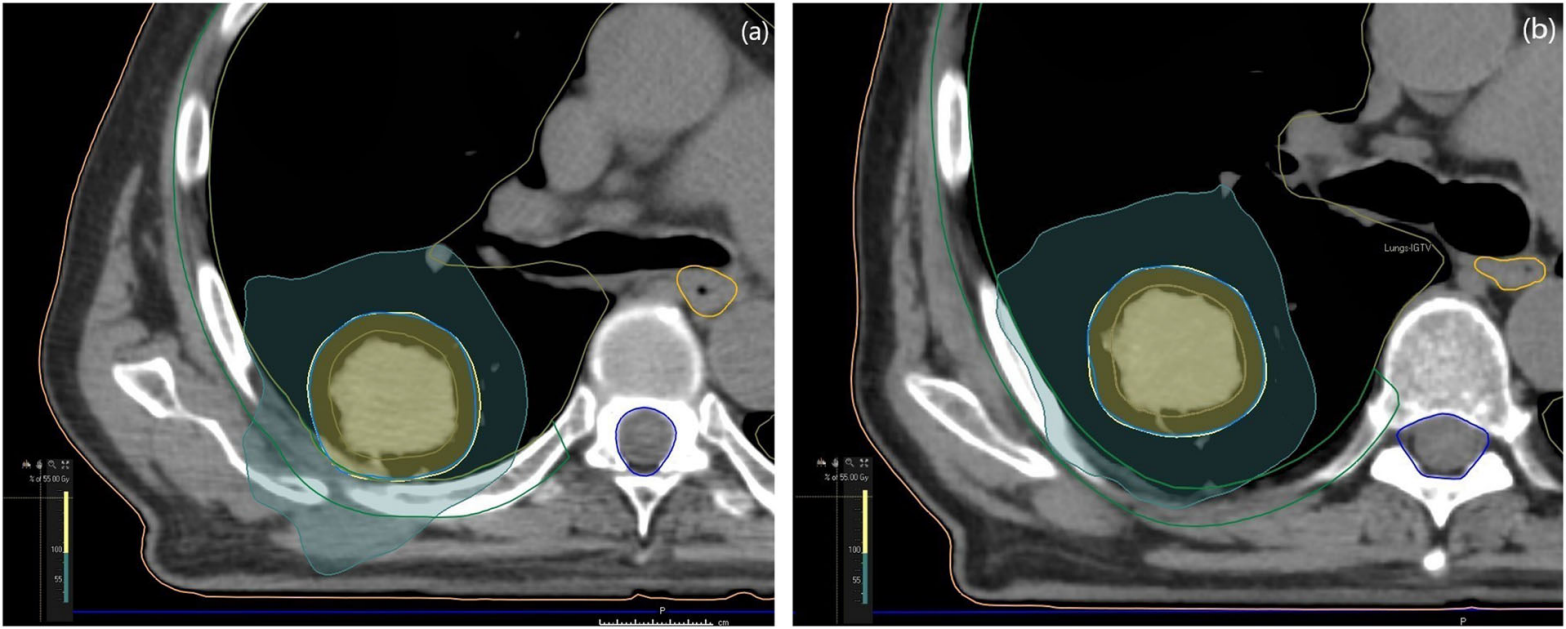
Dorsal view of dose distribution to target in free breathing (a) and deep inspiration breath‐hold (b) for patient 3. Blue isodose 30 Gy, chest wall delineated in green

No significant differences were found in target coverage between the DIBH and FB plans when considering D_98%_ to PTV (Table 1 and Figure 2). The clinical maximum dose did not differ significantly between the two breathing techniques, but in the dosimetric comparison of DIBH and FB, DIBH showed significantly lower doses in all measured volumes except the spinal canal and the esophagus (Table 1). The lung volume in DIBH treatment plans was significantly larger (42%) than that in FB, and the chest wall volume receiving >30 Gy was significantly lower in DIBH than in FB (Table 2). The chest wall and target for patient 6 are shown in Figure 3.

**TABLE 2:** Volume size comparison of free breathing and DIBH in VMAT‐plans

	FB		DIBH			
Parameter	Median	Range	Median	Range	Number of volumes	p‐value
Target						
PTV (cm^3^)	32.28	13.64–72.89	23.46	9.06–61.52	16	<0.01
Conformity index	0.89	0.85–0.92	0.88	0.85–0.91	13	<0.01
OAR						
Lungs (cm^3^)	4282.64	2331.17–7656.70	6087.40	4451.55–9911.26	13	<0.01
Lungs‐GTV V_20Gy_ (%)	3.70	0.47–6.70	2.17	0.45–15.87	13	0.02
Chest wall V_30Gy_ (cm^3^)	12.71	2.17–56.91	10.06	0.15–29.32	14	0.01

Abbreviations: DIBH, deep inspiration breath‐hold; FB, free breathing; GTV, gross tumor volume; OAR, organs at risk; PTV, planning target volume; V_20Gy_, organ volume receiving > 20 Gy; V_30Gy_, organ volume receiving > 30 Gy.; VMAT, volumetric‐modulated arc therapy.

## 4 DISCUSSION

In this study, the implementation of DIBH using modern FFF VMAT techniques in SBRT lung cancer patients was evaluated. The target coverage and OAR doses for both the FB and DIBH plans were assessed. This clinical study found that 20 of 21 lung cancer patients were able to perform respiratory training and hence were eligible for DIBH.

Recent studies have highlighted the ongoing development of the DIBH technique for lung cancer treatment. A study by Josipovic et al.^22^ showed high patient compliance in voluntary DIBH in both CT simulation and RT over 33 fractions. Naumann et al.^23^ showed patient compliance in a small cohort of three patients with lung cancer who underwent SBRT. Several studies have shown DIBH compliance in CT simulation, but the patients were treated with FB.^12^, ^24^ Other studies have shown similar results mainly based on assisted DIBH^13^, ^25^, ^26^ and older treatment techniques.

The lung volume in DIBH compared to that in FB increased by 42%. This is close to the increase previously found in breast cancer patients,^27^ who are generally presumed to have superior lung capacity since their disease does not affect the lungs. Patients included in this study had ECOG status ≤2, and their performance status may have had a positive impact on their ability to hold their breath. Previous research has shown a comparable increase in lung volume in treating NSCLC, showing that these results are representative of the patient group.^11^, ^12^ Giraud et al.^28^ showed an increase in lung volume of only 26%, but a large proportion of the participants were gated with assisted breath‐hold methods, which resulted in poorer lung volume increase than that achieved with voluntary DIBH.^29^, ^30^

The mean estimated beam‐on‐time was lower for DIBH compared to FB, but repeated breath‐hold increased the treatment time overall. The SBRT patients in this study required a time slot of 20 min for DIBH compared to 10 min for the patients treated with FB. This additional time will result in extra costs for the clinic and may reduce the overall availability of RT. The economics of modern equipment for tracking respiratory signals, treatment delivery, and image guided radio therapy (IGRT) may also affect an institution's ability to implement the DIBH technique for lung cancer patients.

The DIBH plans resulted in a significant reduction in the dose to the chest wall compared to FB. Chest wall pain and rib fractures are correlated with the dose per fraction and should be taken into account in SBRT.^31^ A significant decrease of 21% was found in the dose to the chest wall overall, represented by V_30Gy_, for DIBH in comparison with FB. Individually, two of the patients in this study would have received 55 Gy in five fractions in FB due to a lesion in close relationship to the chest wall (for example, Figure 3). These considerations were made to prevent chest wall toxicity. This decrease in chest wall dose is thought to be due to the lesion separating from the chest wall when inflating the lungs. Jaccard et al.^32^ found a reduction in the chest wall dose as the lesion separated from the chest wall in DIBH. The study included only four patients who were eligible for DIBH. Pettersson et al.^33^ found 13 rib fractures in seven of 33 patients treated with SBRT using 45 Gy in three fractions at a median of 29 months after treatment. The risk of radiation‐induced rib fracture following SBRT was related to a high dose of 2 cm^3^ of the rib. Little research exists on this topic, and it needs be investigated further.

As expected, there is significant overall reduction in target size in favor of DIBH. Increasing the volume of the lungs will reduce pressure inside the lungs; therefore, the reduction in target size may be attributable to the absence of motion artifacts in DIBH CT images. Several studies have shown a reduction in the target size when implementing DIBH, either voluntarily or assisted.^11^, ^12^, ^23^, ^28^, ^34^ The target coverage was maintained since there were no significant differences in D_98%_ to CTV and PTV. These findings indicating similar dose coverage are consistent with the results of previous studies.^24^ We found a significant reduction in CI in DIBH in comparison with FB. A possible explanation for these findings might be that the significant reduction in target sizes was followed by well‐known difficulties in achieving optimal conformity due to small target volumes.^35^ An MLC size <5 mm could have resulted in more conformal dose distributions.

Healthy lung tissue received a significantly lower dose in DIBH than in FB, which could reduce the possibility of side effects such as pneumonitis and fibrosis. There was a 29% reduction in the mean lung dose (MLD) when DIBH was applied. This is likely related to a significant increase in lung volume when the lungs are inflated. Josipovic et al.,^11^ Persson et al.,^12^ and Ottosson et al.^24^ all reported reductions of approximately 20% in MLD when applying DIBH. FFF VMAT was applied in this study, which reduces the scattered dose outside the treated field and realizes a sharper dose distribution. Previous studies have shown that the overall dose to healthy lung tissue decreases in DIBH in conventional fractionation, regardless of the DIBH approach used.^11^, ^13^, ^24^, ^25^, ^28^, ^36^

Significant reductions of 40% and 35%, respectively, in the mean and near maximum doses (D_2%_) to the heart were achieved. Several studies have shown decreased doses to the heart, implying that DIBH shows superior OAR sparing than FB.^12^ The patients included in this study had lung lesions located in lung tissue, and only one patient had lymph nodes as a part of the target. The dose to the heart is highly correlated with the location of the target, and no patients had lesions close to the heart in this study.

Intrafraction organ motion possibly contributed to a suboptimal match on IGRT in one patient who was originally eligible for DIBH but subsequently underwent conversion to FB treatment. The lesion was located in the lower left lobe, and the stomach was considered too close to the target in repeated CBCT scans. The fractionation was altered from 15 Gy × 3 to 4 Gy × 7 to spare the stomach from toxicity. The same patient was treated with DIBH for the second target in the opposite lung without any challenges. This might indicate that involuntary intrafractional organ motion, rather than the patient's compliance with the DIBH technique, might have been an issue. Fasting before treatment may have a positive influence on left‐sided lower lobe lung lesions, since it can cause the stomach to be smaller and less active. The relevance of fasting in RT for left lower lobe lung lesions requires further investigation. In general, research has shown small intrafractional deviations in tumor position for lung lesions,^22^, ^23^, ^37^, ^38^ with some cases showing larger variations.

One limitation of this study was the relatively small number of patients who underwent SBRT. The uncertainty in intrafractional motion was included in the PTV margins derived from previous data, but this study did not perform multiple breath‐holds to evaluate each lesion's positional variation, as recommended by Josipovic et al.^22^ Another limitation was that the results were based on dose estimation at the time of the planning CT scan; inhaled volumes can differ during the actual radiotherapy course. Variations in the manner in which patients performed their breath‐hold during the treatment sessions were not accounted for, but repeated imaging was performed before treatment was initiated.

## 5 CONCLUSION

The findings of this study suggest that with contemporary techniques and high‐end equipment, DIBH FFF VMAT can be feasibly performed with high patient compliance in SBRT lung cancer treatment. The DIBH technique allows for target size reduction while maintaining target coverage, and the lower chest wall doses with DIBH can ensure that more patients are candidates for SBRT. DIBH significantly reduces doses to the heart, lungs, and chest wall in lung cancer SBRT. DIBH will not increase beam‐on time; however, DIBH will increase delivery time and may be a disadvantage in clinics with limited resources. A protocol to manage intrafractional deviation could be introduced to mitigate under‐dosage of lesions.

## CONFLICT OF INTEREST

The authors declare no conflict of interest.

## AUTHOR CONTRIBUTIONS

S.T.M., C.L. and C.J. conceived and designed the study. S.T.M., C.L. and J.A.L. were involved inplanning and supervised the study. S.T.M. drafted the manuscript supervised by C.J., and C.J. wrote the final version of the manuscript, while C.L. and J.A.L. contributed with critical feedback.

## Supporting information

Supporting information.
