# Supplementary material for: Clinical experience of volumetric‐modulated flattening filter free stereotactic body radiation therapy of lesions in the lung with deep inspiration breath‐hold
Source: J Appl Clin Med Phys. 2022 Jul 22;23(9):e13733. doi: 10.1002/acm2.13733 (PMC9512343; doi:10.1002/acm2.13733)
Supplement: Supplementary file 1 — Supporting information. [file ACM2-23-e13733-s001.pdf]

SUPPLEMENTARY FILES

Table

Clinical goals in stereotactic body radiotherapy, 15Gy x 3

Structure	Clinical Goal	Priority
PTV	$D_{99\%}$ > 45.0 Gy<br>CI > 0.88 at 45Gy	High<br>Low
Spinal canal	$D_{0.00cc}$ < 21,9 Gy<br>$D_{1.20cc}$ < 12.3 Gy	High<br>High
Trachea	$D_{0.00cc}$ < 30.0 Gy<br>$D_{4,00cc}$ < 15.0 Gy	High<br>High
Bronchus main	$D_{4,00cc}$ < 15.0 Gy<br>$D_{0.00cc}$ < 30.0 Gy	High<br>High
Esophagus	$D_{0.00cc}$ < 25,2 Gy<br>D_{5cc} < 17,7 Gy	Medium<br>Medium
Great vessel	$D_{10,00cc}$ < 39.0 Gy<br>$D_{0.00cc}$ < 45.0 Gy	Medium<br>Medium
Heart	$D_{0.00cc}$ < 30.0 Gy<br>$D_{15,00cc}$ < 24.0 Gy	Medium<br>Medium
Chest wall	V_{30Gy} < 30 cm^3	Low
Contralateral lung	$V_{4,50Gy}$ < 26 %	Low

	D_{mean} < 3.6	Low
External	$D_{2,00cc}$ < 63.0 Gy<br><br>$D_{0.00cc}$ < 67.5 Gy	Low<br><br>Low
Ribs	$D_{2,00cc}$ < 27 Gy<br><br>$D_{0.00cc}$ < 53.8 Gy	Low<br><br>Low
Skin	$D_{10,00cc}$ < 22.5 Gy<br><br>$D_{0.00cc}$ < 24.0 Gy	Low<br><br>Low

Abbreviations: CTV, clinical target volume; CI, Conformity index; D_{15cc}, maximum dose administered to a 15cm^3 volume; D_{5cc} , maximum dose administered to a 5 cm^3 volume; $D_{0,00cc}$, maximum dose administered to a 0,00cm^3 volume; $D_{1,20cc}$, maximum dose administered to a 1,20cm^3 volume; $D_{4,00cc}$, maximum dose administered to a 4,00cm^3 volume; $D_{5,00cc}$, maximum dose administered to a 5,00cm^3 volume; $D_{10,00cc}$, maximum dose administered to a 10,00cm^3 volume; $D_{15,00cc}$, maximum dose administered to a 15,00cm^3 volume; $D_{2\%}$, maximum dose administered to 2% of volume; $D_{99\%}$, dose to 99% of the target volume; D_{mean}, mean dose, GTV, gross tumour volume; PTV, planning target volume; V_{20Gy}, organ volume receiving > 20 Gy; V_{30Gy}, organ volume receiving > 30 Gy; $V_{4.5Gy}$, organ volume receiving > 4.5 Gy.
